# Supplementary material for: Dynamic beam steering with all-dielectric electro-optic III–V multiple-quantum-well metasurfaces
Source: Nat Commun. 2019 Aug 13;10:3654. doi: 10.1038/s41467-019-11598-8 (PMC6692380; doi:10.1038/s41467-019-11598-8)
Supplement: Supplementary file 1 — Supplementary Information [file 41467_2019_11598_MOESM1_ESM.pdf]

Supplementary Materials for

Dynamic Beam Steering with All-Dielectric Electro-Optic III-V Multiple-Quantum-Well Metasurfaces

Pin Chieh Wu[1,2,*], Ragip A. Pala[1], Ghazaleh Kafaie Shirmanesh[1], Wen-Hui Cheng[1], Ruzan Sokhoyan[1], Meir Grajower[1], Muhammad Z. Alam[1], Duhyun Lee[1,3], and Harry A. Atwater[1,4,*]

[1]Thomas J. Watson Laboratory of Applied Physics, California Institute of Technology, Pasadena, California 91125, USA

[2]Department of Photonics, National Cheng Kung University, Tainan 70101, Taiwan

[3]Samsung Advanced Institute of Technology, Suwon, Gyeonggi-do 443-803, South Korea

[4]Kavli Nanoscience Institute, California Institute of Technology, Pasadena, California 91125, USA

Corresponding authors: Pin Chieh Wu (pcwu@gs.ncku.edu.tw); Harry A. Atwater (haa@caltech.edu)

Supplementary Note 1

Analysis of reflectance and phase modulations by resonant MQW heterostructures

To experimentally identify the optimal operation wavelength for observation of tunable amplitude and phase modulations, we fabricate a number of planar DBR/MQW/PMMA/Au heterostructures, which support high-Q Fabry-Pérot resonances (see Supplementary Fig. 1b). The thickness of the Au layer is 35 nm. The spectral position of the high-Q resonances supported by these planar heterostructures can be controlled by changing the thickness of the PMMA layer. The change of the real part of the MQW refractive index (Δn) can be evaluated by examining the shift of the resonant wavelength under applied bias. On the other hand, the change of the imaginary part of the MQW refractive index (Δk) can be evaluated by studying the change of the full width at half maximum (FWHM) of the resonance while applying bias. To fairly evaluate the index change at different wavelengths, we utilize the same resonant mode (i.e. the first Fabry-Pérot cavity resonant mode), which can be gradually shifted by changing the thickness of the PMMA layer in the DBR/MQW/PMMA/Au heterostructure. Supplementary Fig. 1c shows the measured reflectance spectra for different thicknesses of the PMMA layer. It is obvious that the first Fabry-Pérot resonant mode exhibits a red shift when the thickness of the PMMA layer is increased. The second and third Fabry-Pérot resonant modes start showing up when the thickness of the PMMA layer exceeds 210 nm. Since in the case of 438 nm-thick PMMA layer, the first Fabry-Pérot resonant mode shifts out of spectral window of our interest, we analyze the optical modulation with applying bias only in the first six cases shown in Supplementary Fig. 1c (that is, for the thicknesses of the PMMA layers of 0 nm, 22 nm, 37 nm, 167 nm, 210 nm, and 250 nm).

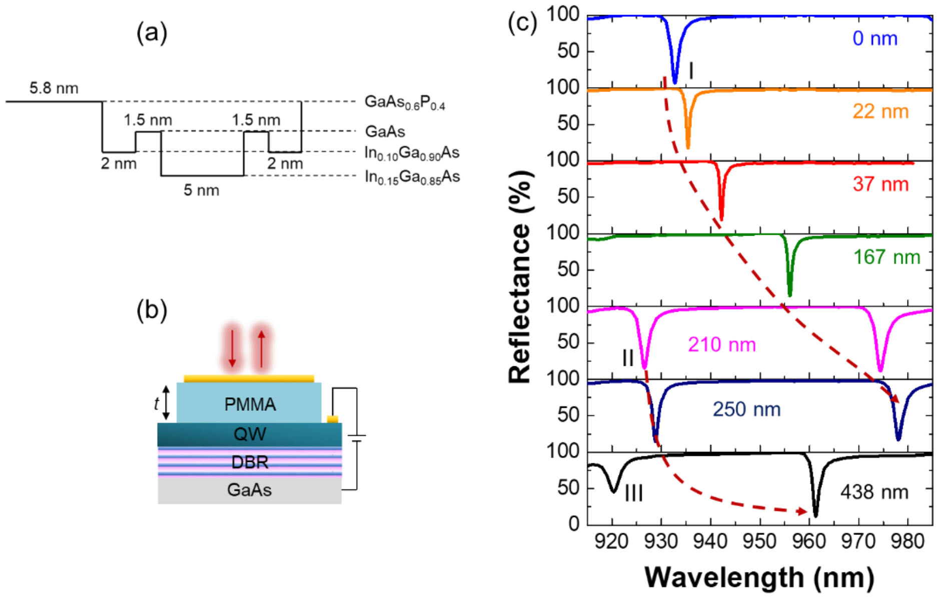

Supplementary Figure 1. (a) Schematic of material combination for the reconstruction of MQW. (b) Schematic of the planar MQW heterostructure. A dielectric PMMA layer is sandwiched between an Au film and a layer of MQWs. The thickness of the PMMA film *t* can be varied during fabrication. (c) Measured reflectance spectra of planar DBR/MQW/PMMA/Au heterostructures for different thicknesses of the PMMA layer. In (c), the legend indicates the thickness of the PMMA layer.

Supplementary Fig. 2 shows the measured reflectance spectra of planar DBR/MQW/PMMA/Au heterostructures for different applied biases. Each subfigure of Supplementary Fig. 2 corresponds to a different thickness of the PMMA layer *t*, which is identified in the legend of the subfigure. Each curve shown in each subfigure of Supplementary Fig. 2 corresponds to the reflectance of the first Fabry-Pérot resonant mode for the applied bias identified in the legend of Supplementary Fig. 2f. Our measurements show stronger amplitude modulation and larger wavelength shifts at shorter wavelengths.

To gain a deeper insight, we use the data displayed in Supplementary Fig. 2 to extract the measured bias-induced wavelength shift of the first Fabry-Pérot resonance supported by the planar DBR/MQW/PMMA/Au heterostructure (see Supplementary Fig. 3). In Supplementary Fig. 3, the wavelength shift $\Delta\lambda$ is defined as an amount of the spectral shift in the resonance position when the bias is changed from 0 V to –10 V, that is, $\Delta\lambda = \lambda_{-10V} - \lambda_{0V}$. Supplementary Fig. 3 also displays a bias-induced variation of the FWHM of the considered Fabry-Pérot resonance as a function of wavelength.

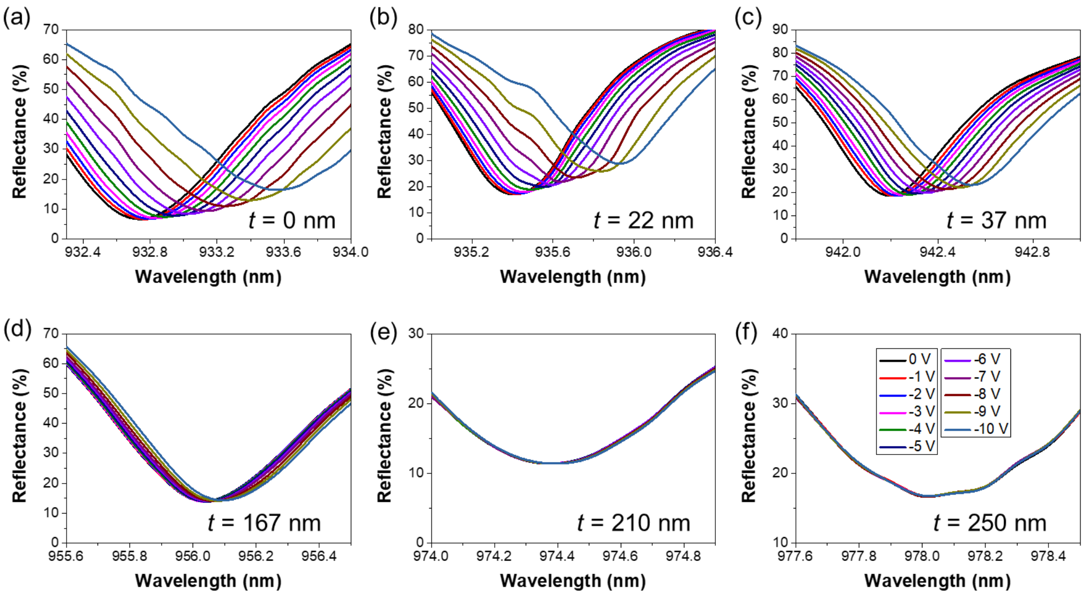

Supplementary Figure 2. Measured reflectance spectra of DBR/MQW/PMMA/Au heterostructure for different PMMA thicknesses under different applied bias voltages.

In Supplementary Fig. 3, the FWHM difference is defined as a change in FWHM when the applied bias changes from 0 V and –10 V. We observe that both the wavelength shift $\Delta\lambda$ and the change in FWHM adopt larger values at shorter wavelengths. Our results are consistent with the analysis described in the prior work[1], which reports the III-V compound MQW design used in our work. Based on the trend shown in Supplementary Fig. 3, we believe that the strongest refractive index modulation occurs at wavelengths very close to the absorption band edge of our quantum wells, which we expect to correspond to the wavelength of ~915 nm.

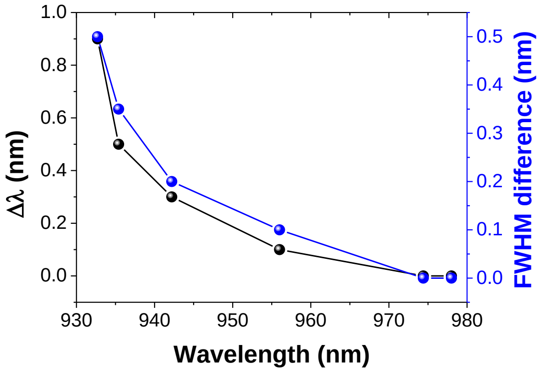

Supplementary Figure 3. Measured wavelength shifts (black dots) and (b) FWHM difference (blue dots) at the first Fabry-Pérot resonant mode. The presented data is extracted from the curves shown in Supplementary Fig. 2.

Supplementary Note 2
Calculation of multipole decomposition

To further analyze the modes supported by the MQW metasurface, we performed a multipole decomposition analysis using the charge-current expansion framework[2-4]. In our calculations, we account for contributions of electric and magnetic dipoles, quadrupoles, and octupoles. Supplementary Fig. 4 shows results of the radiated electric field intensity contributed from various electromagnetic multipoles. For simplicity, only the first three leading terms are shown. As expected from the field distribution presented in Figs. 2c and 2d, the magnetic octupole (which can be interrupted as a high-order Mie resonance) plays a significant role in the optical response under *x*-polarized illumination.

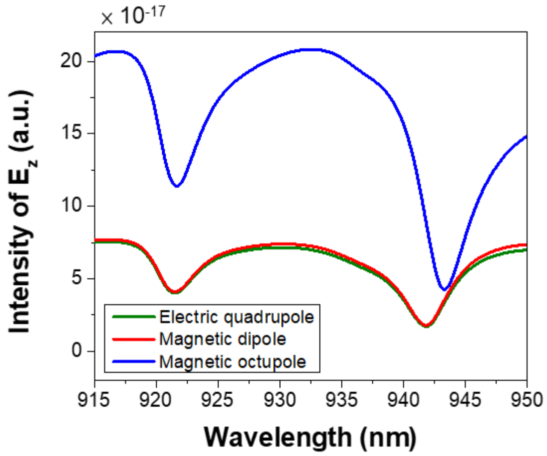

Supplementary Figure 4. Simulated *z*-component of far-field electric intensity for electromagnetic multipoles. For simplicity, only the first three leading terms are presented.

Asymmetry issue in hybrid Mie-GM resonant mode

To study how asymmetric slit positioning affects the optical performance of the device (which is originally caused by the misalignment of the electron beam lithography process), we calculated the reflectance for different values of the offset parameter *s* (see Supplementary Fig. 5a). The offset parameter *s* is defined as the spatial displacement of center of the topmost partially etched slits with respect to the underlying MQW slab. Since the partially etched slits act as a light coupler and assist in excitation of guide-mode (GM) resonance, the generation of hybrid Mie-GM resonance is not significantly influenced by the structural asymmetry. Our simulations show that the hybrid Mie-GM resonance exists in all three cases, $s = 0$ nm, 40 nm, and 80 nm. However, this resonance blue-shifts when the spatial offset (*s*) is increased. To observe significant optical modulation, we need to ensure such high-quality resonance is spectrally located in the wavelength region where the utilized MQW exhibits the largest index modulation (that is, from 915 nm to 920 nm). Supplementary Fig. 5(b) indicates that the largest acceptable spatial offset *s* is about 80 nm (it is about 50 nm in our first fabricated MQW metasurface).

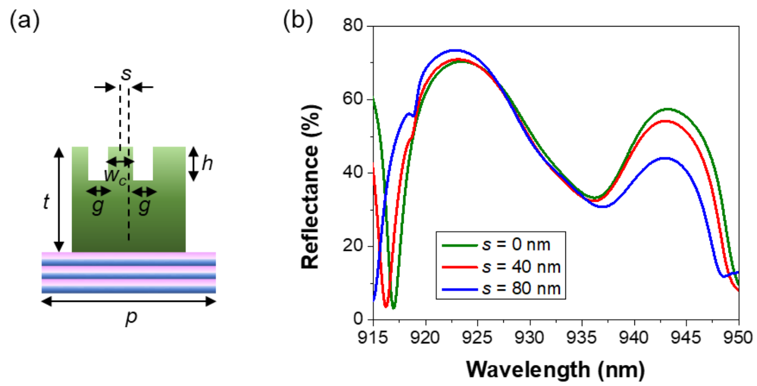

Supplementary Figure 5. (a) Schematic for the hybrid Mie-GM resonant metasurface. The unit element dimensions are defined as follows: w_c = 180 nm, g = 100 nm, t = 1230 nm, h = 40 nm, and p = 910 nm. The parameter s is defined as the spatial offset of the partially etched slits from the center of the underlying MQW slab. (b) Simulated reflectance spectrum for different spatial offsets s.

Supplementary Note 3

Origin of the resonant modes in MQW resonators

To investigate the origin of each resonant mode in the MQW resonators, here we perform the simulations for the MQW structures, which are comprised of the partially etched double-slits (see Supplementary Fig. 6a). Note that in Supplementary Fig. 6a, the air gap between the resonant elements, which is otherwise present in the fabricated structures, is absent. Supplementary Fig. 6b shows the simulated reflectance spectrum, in which two resonant features are observed. The spatial distribution of the x-component of the electric field intensity E_x shows that the MQW layer supports a Fabry-Pérot resonance within the MQW layer at the shorter-wavelength resonant dip (see Supplementary Fig. 6c). Since E_x is primarily enhanced in the spatial region between the groups of double-slits, this Fabry-Pérot cavity resonance will be strongly suppressed when the fully etched air gap is present (see Supplementary Fig. 7a). However, we still observe strong filed enhancement within the fully etched air gap, which is mainly from the near-field interaction between MQW slabs (see Supplementary Fig. 7a). More interestingly, the double-slit structure can assist in exciting a guided-mode resonance (GMR), resulting in the large E_z intensity at the topmost interface of the MQW structure (see Supplementary Fig. 6d). Concurrently, we observe a quite signifcant E_z intensity inside the MQW layer (see Supplementary Fig. 6d). On the other hand, for the longer-wavelength resonant dip, we observe that the double-slit

structure acts as a coupler, which effectively guides the incident light into the MQW layer, yielding a strong field confinement in the slits, as shown in Supplementary Fig. 6c. As a result, a Fabry-Pérot-like resonance can still survive when the MQW layer is truncated (see Supplementary Fig. 7b). This light coupler can also weakly convert the incident electric field from *x*- to *z*-component, as shown in Supplementary Fig. 6d.

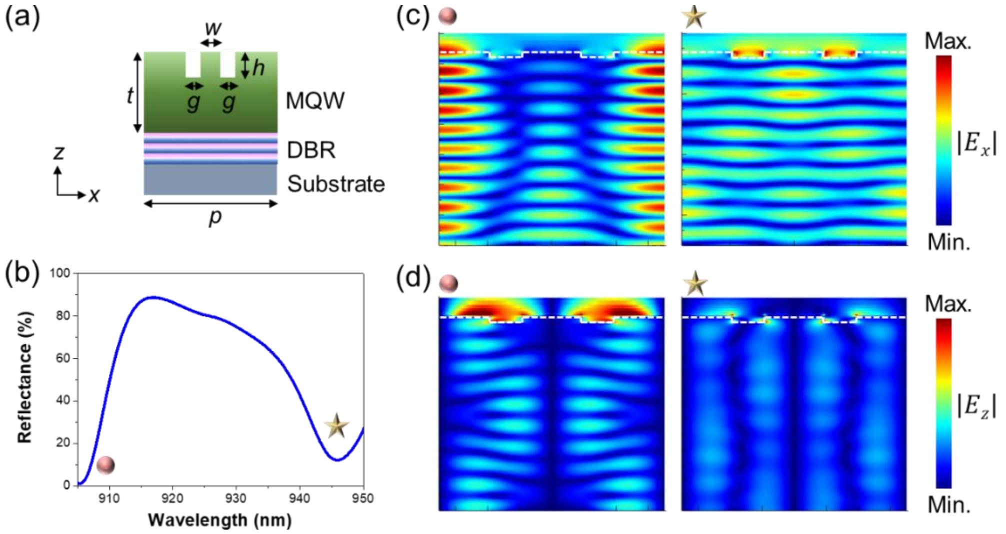

Supplementary Figure 6. (a) Schematic for the simulated structure. The unit element dimensions are defined as follows: w = 180 nm, g = 100 nm, t = 1230 nm, h = 40 nm, and p = 910 nm. (b) Simulated reflectance spectrum of the double-slit structure illustrated in (a). (c) and (d) show the spatial distributions of the E_x and E_z intensities at the wavelengths corresponding to the spectral dips. The incident polarization is along *x*-axis.

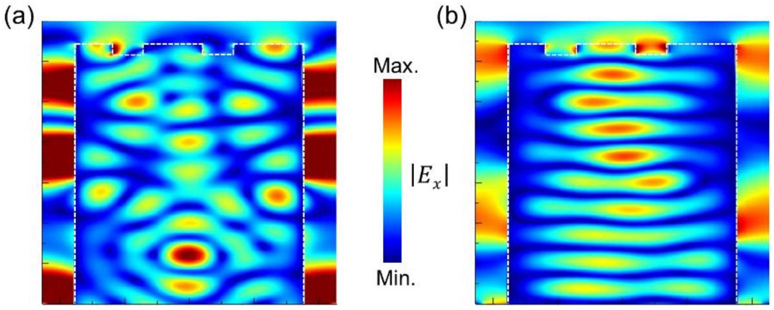

Supplementary Figure 7. Simulated *x*-component of electric field intensity in MQW resonator at a wavelength of (a) 915.9 nm and (b) 936.3 nm, respectively.

Supplementary Note 4
Mode splitting in the hybrid Mie-GM resonant metasurface

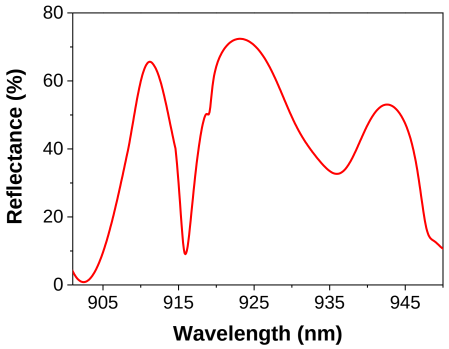

Supplementary Figure 8. Simulated reflectance spectrum of a Mie-GM resonant metasurface under an *x*-polarized normal illumination. Here, we extend the wavelength range to shorter wavelengths as compared to the wavelength range over which we performed our simulations. The structural parameters are identical to the ones described in Fig. 2.

Supplementary Note 5
Optical setup for measurement of reflectance spectrum

To optically characterize the reflectance of the MQW metasurface, we utilized a coherent NIR laser beam (Toptica Photonics CTL 950) as a light source and a power meter as a detector (Thorlabs PM100D), as shown in Supplementary Fig. 9. An uncollimated white light source from a halogen lamp is used to visualize the sample surface. When measuring the reflectance spectra, the laser beam was focused using a long working distance objective with 10× magnification and 0.28 numerical aperture.

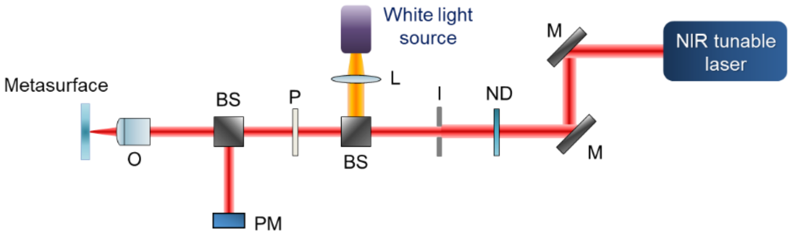

Supplementary Figure 9. Schematic of optical setup used for spectral measurement. M: mirror; ND: neutral density filter (Thorlabs NDC-50C-4M); I: iris; L: lens; P: linear polarizer (Thorlabs LPNIR100-MP); BS:

beam splitter (Thorlabs CCM1-BS014); O: objective (Mitutoyo 10× magnification with 0.28 numerical aperture); PM: power meter.

Measured absolute reflectance modulation

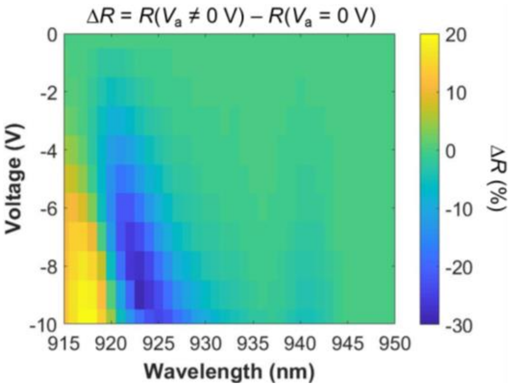

Supplementary Figure 10. Measured absolute reflectance modulation ΔR of the hybrid Mie-GM resonant metasurface.

Supplementary Note 6

Influence of oblique illumination on the optical diffraction

Because of the slight difference in structural period (910 nm) and laser wavelength (917 nm), optical diffraction can influence the far-field radiation pattern when the incident angle is non-zero. To clarify this point, we performed numerical simulations of the far-field radiation patterns for the MQW metasurface at 0 V with different angles of incidence. As shown in Supplementary Fig. 11a, we found that strong optical diffraction appears even when the incident angle is 5°. However, those diffracted beams' intensities are high compared as to the intensity of the zero-order beam, which is not observed in our measurements. This can be attributed to two different reasons; first, their diffraction angles are too large to be collected (based on the numerical aperture of the objective we used, the largest angle collected is about 16°) and second, the incident angle is almost zero in the real case. To experimentally eliminate this effect, we intentionally slightly defocused the laser beam onto the MQW metasurface when performing the far-field radiation measurements to minimize the incident angle.

To further verify the influence of non-zero incident angle on the far-field radiation pattern, we performed other simulations which numerically demonstrate the active switching of the first-order diffracted beam at different angles of incidence. As shown in Supplementary Fig. 11b, the intensity of the first-order diffracted beams are much higher as compared with the specularly reflected beam when the incident angle is greater than 5°, which is in conflict with our measurement results shown in Figs. 4d and 4e. As a result, we conclude that the MQW metasurface is under almost normal illumination ($0^\circ \leqq \theta_{in} \leqq 5^\circ$), and the non-zero incident angle caused optical effect is fairly small in the real case. It is worth noting that the first-order diffracted beams can only be observed when electrical bias is applied, indicating that the demonstration of active switching of first-order diffracted beam is still valid even when the MQW metasurface is under oblique illumination.

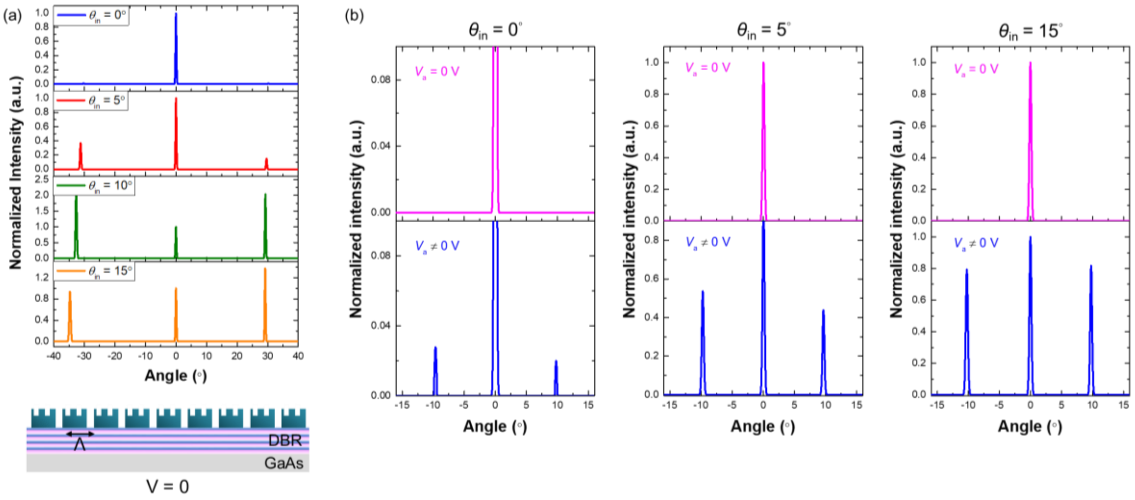

Supplementary Figure 11. (a) Simulation of far-field radiation patterns under oblique illumination without electrical bias. Strong diffraction can be observed when the incident angle is greater than 5°. (b) Simulation of active switching of first-order diffraction for the case of normal (left panel) and oblique (middle and right panels) illumination. Overall, the diffracted beams show much stronger intensity when incident angle is greater than 5°. The incident wavelength is fixed at 917 nm.

Supplementary Note 7

Theoretical analysis and experimental measurement of high-speed reflectance modulation

We first theoretically estimate the modulation speed of the MQW metasurface. The conductivity of the p-GaAs (with doping level of 10^{19} cm^{-3}):

$$\sigma = qp\mu_p = 1.6x10^{-19} \cdot 1x10^{19} \cdot 67 \cong 107 \ [\frac{1}{\Omega \cdot cm}] \qquad (1)$$

where q is the electron charge, p is the carrier concentration, $\mu_p \cong 67$ $[\frac{cm^2}{V \cdot s}]$ is the hole mobility[5]. The RC delay of the MQW system is

$$RC = \frac{L}{\sigma d_1 w_{MQW}} \frac{\varepsilon_0 \varepsilon_r L w_{MQW}}{d} = L^2 \frac{\varepsilon_0 \varepsilon_r}{\sigma d_1 d} = 1.816x10^{-9} \ [s] \qquad (2)$$

where L = 100 μm and w_{MQW} is the length and width of the hybrid Mie-GM resonant structure respectively, d_1 is the thickness of p-GaAs, d is the thickness of MQW, ε_0 is the permittivity in vacuum, and ε_r = 13.5 is the dielectric constant[6, 7] of the MQW. Therefore, the estimated highest modulation frequency f is:

$$f = \frac{1}{2\pi RC} = 87.66 \ [MHz] \qquad (3)$$

To experimentally evaluate the modulation speed of our MQW metasurface, an AC electrical bias with frequencies of 0.1 MHz and 1 MHz is applied to the sample and a high-speed InGaAs detector is used to detect the temporal amplitude response (see Supplementary Fig. 12a). As shown in Supplementary Fig. 12b, high-speed amplitude modulation with 0.1 MHz frequency bandwidth is performed. Modulation speed as high as 1 MHz is also demonstrated, which is on the order of the theoretical expectation. The difference between the theoretical calculation and the measured response is likely due to the deviation between parameters (conductivity, dielectric constant, *etc*) used in calculations and those of the real sample as well as the fact that the contact resistance is not taken into account in the theoretical estimation. In principle, the quantum confined Stark effect can yield the devices operating with GHz modulation speed if the intrinsic RC delay is further minimized, which can be accomplished by reducing the length of resonators, increasing the thickness of p-GaAs, *etc*. Note that the signal is distorted in the case of 1 MHz modulation speed due to the bandwidth limitation of the power amplifier.

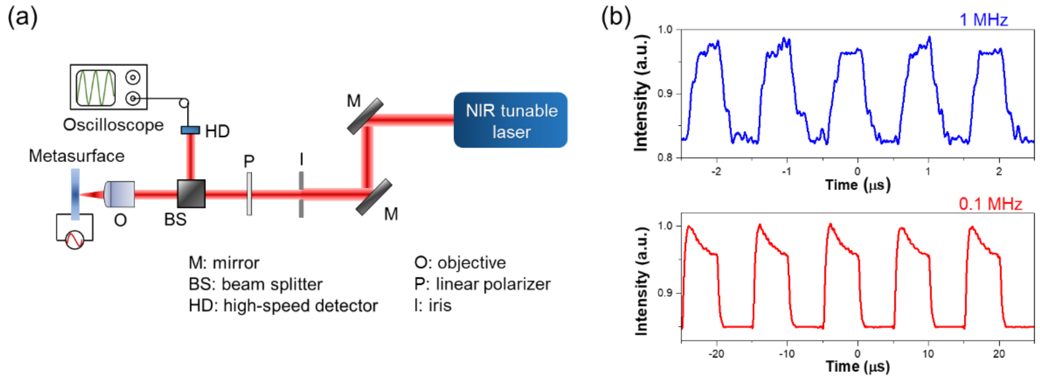

Supplementary Figure 12. Experimental performance of modulation speed measurement in all-dielectric MQW metasurface. (a) Schematic of the optical setup combined with a high-speed detector (Thorlabs DET10C) and an oscilloscope (Tektronix TDS 2001C). The AC electric field is applied from a function waveform generator (Keysight 33220A) combined with a power amplifier (Thorlabs HVA200). (b) The measured results of temporal response of hybrid Mie-GM metasurfaces. Blue curve: 1 MHz, red curve: 0.1 MHz. The wavelength of incident light is fixed at 917 nm.

Supplementary Note 8
Measurement of the far-field radiation pattern

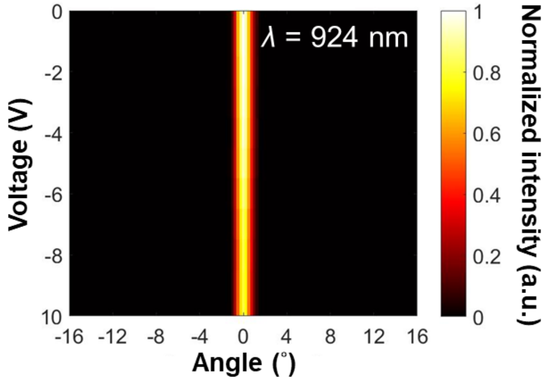

Supplementary Figure 13. Experimental results of far-field radiation intensity profile under different applied biases. No first diffraction order is observed at a wavelength of 924 nm even when the applied bias voltage is -10 V.

Supplementary Note 9
Optical response and modulation of the beam steering metasurface

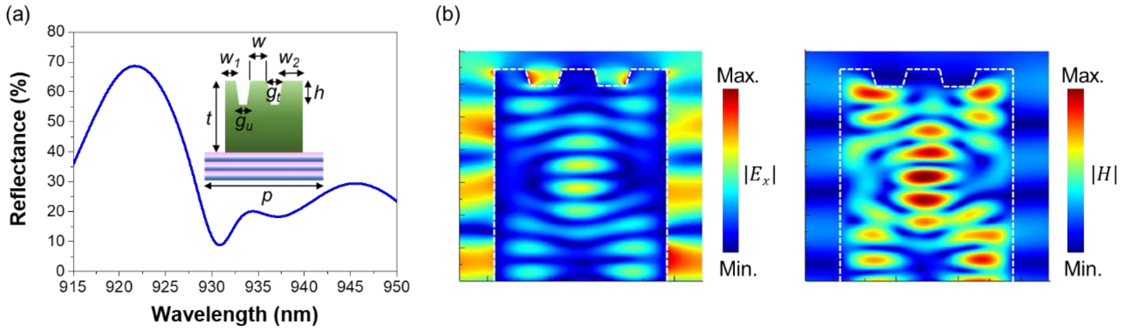

Supplementary Figure 14. (a) Simulated reflectance spectra of the second hybrid Mie-GM resonator array for different applied bias voltages. The incoming light is polarized perpendicularly to the MQW stripes. Structural parameters: w_1 = 100 nm, w_2 = 120 nm, w = 110 nm, g_t = 110 nm, g_u = 90 nm, t = 1230 nm, and h = 80 nm. The periodicity p is 780 nm. (b) Simulated electromagnetic field profiles at a wavelength of 917 nm.

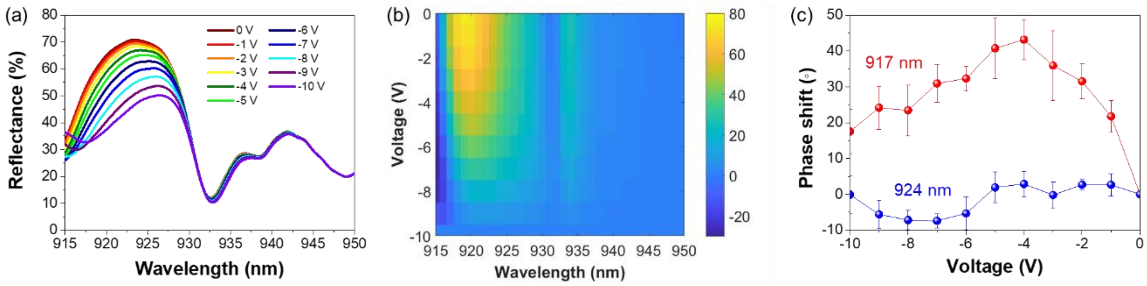

Supplementary Figure 15. (a) Measured reflectance spectra of the second Mie resonator array for different applied bias voltages. The incoming light is polarized perpendicularly to the MQW stripes. (b) Measured relative reflectance of the second hybrid Mie-GM resonant metasurface as a function of wavelength and applied voltage. We consider the wavelength range from 915 nm to 925 nm with a step of 1 nm. Here, the reflectance of resonator under a -10 V bias is utilized as the reference. (c) Measured phase modulation at two different wavelengths. Red: 917 nm, blue: 924 nm. Each data point corresponds to an average phase shift measured at four different positions on the sample while each error bar indicates the standard deviation of the obtained four data points.

Supplementary Note 10

Simulation of far-field radiation pattern of the beam steering metasurface with varying lattice constant *Λ*

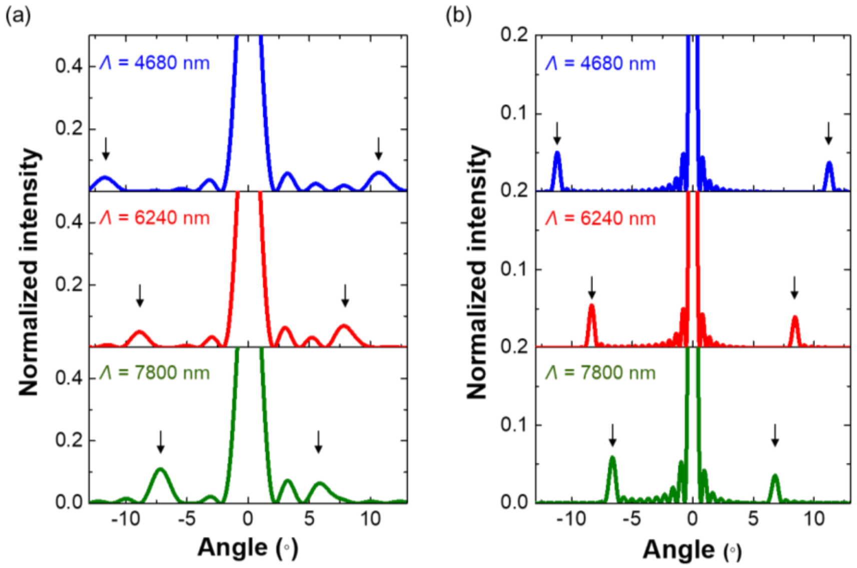

Supplementary Figure 16. Simulated results of far-field beam steering by changing the periodicity of metasurface *Λ*. The total number of metasurface unit element is assumed to be (a) 30 and (b) 120. Black arrows indicate the position of the first diffraction order. Incident wavelength: 917 nm. In order to take the finite aperture effect into account, the top hat is utilized as the illumination condition when processing the far field projection in Lumerical FDTD simulation.

Supplementary Note 11

Measured J-V curves of MQW resonators

Supplementary Fig. 17 shows the measured leakage current density of two MQW resonators. To avoid the dielectric breakdown, we applied a moderate bias ranging from 0 V to -10 V. For both of our samples, the measured current density is on the order of mA/cm^2, which is much lower than the current density values (on the order of of ~kA/cm^2) necessary for observation for carrier-induced refractive index change in GaAs-based III-V semiconductor compounds[8-10].

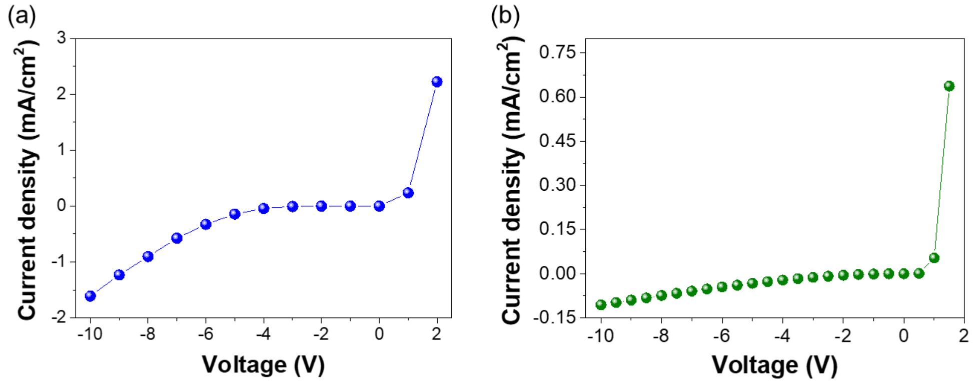

Supplementary Figure 17. Measured J-V curves of the (a) first and (b) second hybrid Mie-GM resonators.

Supplementary Note 12

Material composition and optical index of the MQW sample

(a)

Remarks	Material	Thickness (nm)
Contact	p-GaAs	50
QW/Barrier (60 pairs)	(see Supplementary Fig. 1a)	1230
Spare	$GaAs_{0.6}P_{0.4}$	5.8
Cladding	n-$Al_{0.31}Ga_{0.69}As$	7.6
Etch-stop	InGaP	10
Contact	n-GaAs	20
Etch-stop	InGaP	10
DBR (20 pairs)	n-$Al_{0.90}Ga_{0.10}As$	76.5
	n-GaAs	65
Substrate	GaAs	600,000

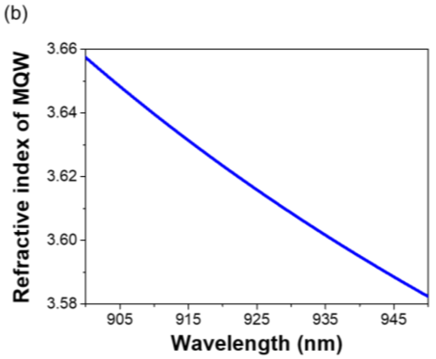

Supplementary Figure 18. (a) Details of material composition of MQW sample used in this work. (b) The real part of refractive index of MQW used in all simulations. The imaginary part of the refractive index was set to a constant of 0.002 for simplicity.

Supplementary Note 13

Improvement of optical performance

To improve the optical performance, the quantum well system has to provide a substantial change in the real part of the refractive index, Δn, to sufficiently shift the resonances, and maintain a small change in the imaginary part of refractive index (absorption), Δk, to enable a sharp resonance within the operating wavelength range. As a

result, the larger the figure of merit, $\Delta n/\Delta k$ of a quantum well, the better optical performance can be achieved in the tunable metasurface. As a proof of concept, we designed a tunable metasurface with an asymmetric coupled quantum well (ACQW) which can possess a larger $\Delta n/\Delta k$ (about 10-18)[11]. As a comparison, the $\Delta n/\Delta k$ of utilized MQW in this work is 1-5 (see Ref. 1). The unit element is also based on the double-slit structure, as shown in Supplementary Fig. 19a. After structural optimization, we found that about 200° phase shift with a Δn of 0.02 can be obtained at a wavelength of 808.8 nm (see Supplementary Fig. 19b). We also numerically study the beam steering functionality using such ACQW-based metasurface, which is realized by varying the periodicity of the metasurface (see Supplementary Fig. 19c). The simulated far-field radiation patterns even show that the intensity of the steered beams are much higher than the intensity of the specularly reflected beam when the utilized QW possesses larger $\Delta n/\Delta k$. These results indeed verify that the optical performance (in particular, directivity, which is defined as the peak intensity ratio between diffracted and mirror reflected beams) of tunable quantum well-based metasurfaces can be significantly improved when the quantum well system exhibits larger $\Delta n/\Delta k$. Since this is a proof-of-concept demonstration, the working wavelength here (λ = 808.8 nm) is slightly different from the one used in the main manuscript. By appropriately choosing a quantum well, we can shift the operation wavelength to the range of interest[12, 13].

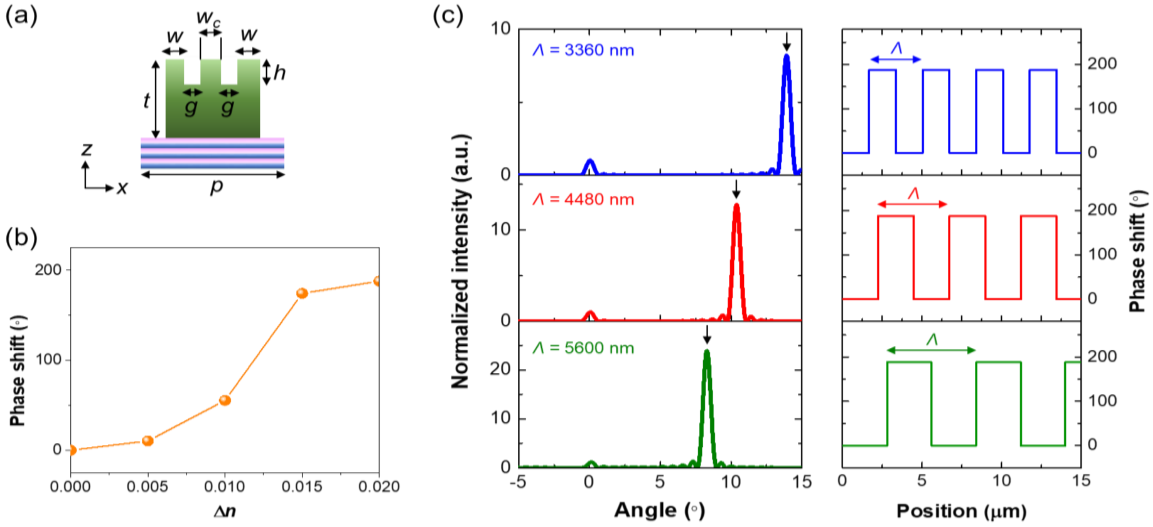

Supplementary Figure 19. Simulated QW resonant metasurface with higher $\Delta n/\Delta k$. (a) A schematic for all-dielectric asymmetric coupling quantum well (ACQW) metasurface. The unit element dimensions are

defined as follows: w_c = 90 nm, w = 60 nm, g = 100 nm, t = 1230 nm, and h = 80 nm. The periodicity p is 560 nm. (b) Simulated phase shift as a function of Δn of an ACQW resonant metasurface under an x-polarized normal illumination. (c) Simulated intensity of the scattered light in the far-field as a function of diffraction angle. The plotted diffracted light intensity is normalized to the light intensity at 0°. Right panel shows the corresponding phase profile for each case. The first-order diffracted beam shows much higher intensity as compared to intensity of the specularly reflected beam. The phase shift and light intensity are plotted for a wavelength of 808.8 nm. Black arrows indicate the position of the first-order diffracted beams. Due to the spatial symmetry, only half of the radiation pattern is presented. The total number of unit elements is set at 120.

Supplementary References

1. Cho Y.-C., Lee Y.-T., Chang-Young P., Byung-Hoon N., Park Y.-H., Ju G.-W., et al., Optical device including three coupled quantum well structure. Google Patents, assignee, US20150286078A1 (2015).
2. Savinov V., Fedotov V. A., Zheludev N. I. Toroidal dipolar excitation and macroscopic electromagnetic properties of metamaterials. *Phys. Rev. B* **89**, 205112 (2014).
3. Wu P. C., Liao C. Y., Savinov V., Chung T. L., Chen W. T., Huang Y.-W., *et al.* Optical anapole metamaterial. *ACS Nano* **12**, 1920-1927 (2018).
4. Zhu A. Y., Chen W. T., Zaidi A., Huang Y.-W., Khorasaninejad M., Sanjeev V., *et al.* Giant intrinsic chiro-optical activity in planar dielectric nanostructures. *Light Sci. Appl.* **7**, 17158 (2018).
5. Casey H. C., Stern F. Concentration-dependent absorption and spontaneous emission of heavily doped GaAs. *J. Appl. Phys.* **47**, 631-643 (1976).
6. Brennan K., Hess K. High field transport in GaAs, InP and InAs. *Solid State Electron.* **27**, 347-357 (1984).
7. Won-Pyo H., Bhattacharya P. K. High-field transport in InGaAs/InAlAs modulation-doped heterostructures. *IEEE T. Electron. Dev.* **34**, 1491-1495 (1987).
8. Dutta N. K., Olsson N. A., Tsang W. T. Carrier induced refractive index change in AlGaAs quantum well lasers. *Appl. Phys. Lett.* **45**, 836-837 (1984).
9. Ozaki S., Adachi S. Spectroscopic ellipsometry and thermoreflectance of GaAs. *J. Appl. Phys.* **78**, 3380-3386 (1995).
10. Jong-In S., Yamaguchi M., Delansay P., Kitamura M. Refractive index and loss changes produced by current injection in InGaAs(P)-InGaAsP multiple quantum-well (MQW) waveguides. *IEEE J. Sel. Top. Quant.* **1**, 408-415 (1995).
11. Hao F., Pang J. P., Sugiyama M., Tada K., Nakano Y. Field-induced optical effect in a five-step asymmetric coupled quantum well with modified potential. *IEEE J. Quantum Elect.* **34**, 1197-1208 (1998).
12. Xu Z., Wang C., Qi W., Yuan Z. Electro-optical effects in strain-compensated InGaAs/InAlAs coupled quantum wells with modified potential. *Opt. Lett.* **35**, 736-738 (2010).
13. Mohseni H., An H., Shellenbarger Z. A., Kwakernaak M. H., Abeles J. H. Enhanced electro-optic effect in GaInAsP–InP three-step quantum wells. *Appl. Phys. Lett.* **84**, 1823-1825 (2004).
